# Non-Toxic Crosslinking of Electrospun Gelatin Nanofibers for Tissue Engineering and Biomedicine—A Review

**DOI:** 10.3390/polym13121973

**Published:** 2021-06-15

**Authors:** Andrea Ehrmann

**Affiliations:** Working Group Textile Technologies, Faculty of Engineering and Mathematics, Bielefeld University of Applied Sciences, 33619 Bielefeld, Germany; andrea.ehrmann@fh-bielefeld.de

**Keywords:** nanofiber blends, water-solubility, polymer complex, polymer blend, crosslinker, genipin, aldehydes, UV-crosslinking, electron beam, photo initiator

## Abstract

Electrospinning can be used to prepare nanofiber mats from diverse polymers, polymer blends, or polymers doped with other materials. Amongst this broad range of usable materials, biopolymers play an important role in biotechnological, biomedical, and other applications. However, several of them are water-soluble, necessitating a crosslinking step after electrospinning. While crosslinking with glutaraldehyde or other toxic chemicals is regularly reported in the literature, here, we concentrate on methods applying non-toxic or low-toxic chemicals, and enzymatic as well as physical methods. Making gelatin nanofibers non-water soluble by electrospinning them from a blend with non-water soluble polymers is another method described here. These possibilities are described together with the resulting physical properties, such as swelling behavior, mechanical strength, nanofiber morphology, or cell growth and proliferation on the crosslinked nanofiber mats. For most of these non-toxic crosslinking methods, the degree of crosslinking was found to be lower than for crosslinking with glutaraldehyde and other common toxic chemicals.

## 1. Introduction

Gelatin is derived from collagen by partial hydrolysis, either by acid processing (type A) or by alkaline or lime processing (type B) [1]. Gelatin is often processed from waste, e.g., from fish skin and bones after filleting [2]. As a natural material, gelatin shows different physical and chemical properties, depending on its origin [3,4,5]. Values typically measured in comparative studies are, e.g., the molecular weight, amino acid composition, gel strength, viscosity, melting point, as well as breaking force, water vapor permeability, or water solubility.

Gelatin’s physical and chemical properties as well as the abundant availability make it interesting for diverse applications in food, cosmetics, and the pharmaceutical industry, but also as a coating of photographic films. Other important areas of application are biotechnology and biomedicine. Especially gelatin-based porous scaffolds are of high interest in tissue engineering [6]. However, here, a challenge occurs due to the water-solubility of gelatin, which necessitates crosslinking after producing the scaffolds. There are different physical methods, such as UV irradiation or dehydrothermal treatment [7,8], chemical methods such as exposure to glutaraldehyde in the form of vapor or liquid [9,10] or to ethylcarbodiimidehydrochlorine (EDC) combined with N-hydroxysuccinimide (NHS) [11,12], or enzymatic treatment by genipin or transglutaminases [13,14]. Generally, the crosslinking method influences the physical and chemical properties of the resulting scaffold [15,16,17].

This is especially important for nanofibrous scaffolds, as they are often produced by electrospinning. The structure of these ultrathin fibers is usually modified by the crosslinking process, in this way also modifying the porosity, mechanical properties, and suitability for cell growth. Here, we give an overview of recent crosslinking techniques for electrospun gelatin nanofiber mats and their suitability for tissue engineering, with the objective to shed light on alternatives to common glutaraldehyde crosslinking, but also making clear which crosslinking degrees can be reached by which method, to allow researchers on this base to decide which process is most suitable for their recent application.

## 2. Electrospun Gelatin Nanofibers—Production and Properties

**Figure 1:**
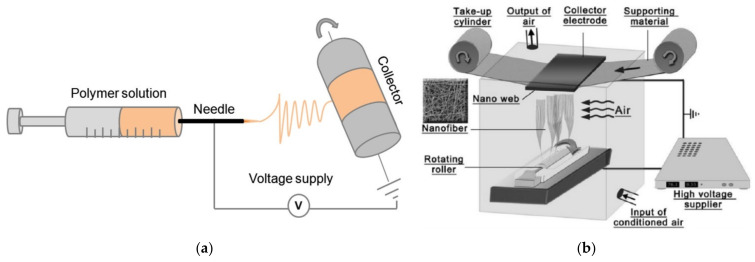
Examples of electrospinning techniques: (**a**) Needle-based electrospinning, from [21], originally published under a CC-BY license; (**b**) wire-based electrospinning, from [22], originally published under a CC-BY license.

Electrospinning can be used to produce nanofiber mats by a relatively simple technique. In brief, a polymer solution or melt is introduced into an electric field that is produced by a high voltage between two electrodes, where the polymer solution or melt is accelerated to the counter electrode and in this way stretched until ultrathin fibers are deposited on the substrate, which usually shields the counter electrode [18,19,20]. Often used techniques are needle-based electrospinning (Figure 1a) [21] and wire-based electrospinning (Figure 1b) [22], while there are diverse other technologies available in the broad range of needleless electrospinning.

Electrospinning gelatin is in principle possible from aqueous solutions. For example, Zhang et al. investigated the influence of the spinning temperature as well as of the solid content in the solution on the morphology of the gelatin nanofiber mat [23]. They dissolved 30–40% gelatin with a molecular weight of 25 kDa in distilled water of 40 °C, varied the temperature of the solution in the syringe during spinning between 30 and 50 °C, and found that the viscosity decreased with temperature and increased with solid content, while the conductivity increased with temperature and with concentration. The average fiber diameter slightly decreased with temperature and strongly increased with gelatin concentration. Crosslinking was performed with EDC/NHS in a ratio of 2.5:1, resulting in reduced weight loss, significantly reduced swelling, and clearly modified stress–strain curves, which also depended strongly on whether they were measured in the dry or in the wet state.

Kwak et al. compared cold fish gelatin nanofibers electrospun from pure water as solvent with acetic acid/water (50:50, *v*/*v*) and 2,2,2-trifluoroethanol (TFE), which are also often used [24]. They found relatively high viscosities and concentrations to be advantageous for the formation of uniform fibers, while reduced values led to beaded fibers or pure beads. The fiber diameters again increased with the gelatin concentration, while flow rate, voltage, and distance between the needle tip and collector did not show a significant impact. Comparing the three different solvents, spinning from distilled water reached the highest productivity, especially for relatively high flow rates around 0.4 mL/h.

Depending on the exact type of gelatin, spinning from pure water is often challenging. Ki et al. reported about this problem and suggested formic acid as a possible solvent [25]. They also found increasing fiber diameters with increasing concentration and no significant impact of the spinning distance on the diameter. From circular dichroism spectra, they concluded that the original helical conformation of the gelatin powder seemed to be transferred into a random coil conformation if electrospinning is performed from formic acid. This visible degradation of gelatin by formic acid is only one example of many reported in the literature, showing an impact of the used solvent on the physical and also chemical properties of the electrospun nanofibers. This may lead to the necessity to take the production process into account when optimizing the crosslinking procedure.

Acetic acid is another possible solvent, as suggested by Gu et al. for needle-based electrospinning of porcine skin gelatin [26]. They used acetic acid of 50–90% concentration and found that higher acid concentrations to lead to bead-free fibers, while also a minimum of 10% gelatin concentration was necessary to avoid beads, which was attributed to the higher viscosity for higher solid content, counteracting the bead-forming surface tension. At the same time, a higher gelatin concentration again increased the average fiber diameter.

As an alternative to acetic acid, Song et al. used acetic acid/ethyl acetate in different ratios and compared both solvents with hexafluoro-2-propanol (HFP), which is an often used but toxic solvent for spinning gelatin nanofibers [27]. They found again a relatively high percentage of acetic acid necessary to avoid beads and the fiber diameter increasing with the gelatin concentration. However, adding ethyl acetate could be used to increase the spinnability and reduce the solvent acidity. In addition, this co-solvent approach resulted in the only sort of gelatin nanofibers that revealed in a differential scanning calorimetric (DSC) analysis an endothermic peak near 220 °C, as the original gelatin powder does. In contrast, the nanofiber mats spun from acetic acid and HFP did not show such a peak anymore, suggesting that these solvents seemed to negatively influence the gelatin structure.

Varying the gelatin concentration in TFE, Huang et al. also found an increasing fiber diameter with increasing solid content between 5% and 15% [28]. In addition, they investigated the mechanical properties of the electrospun nanofiber mats and suggested finer fibers for higher tensile modulus and ultimate tensile strength, while they also recognized a negative effect of beads on these mechanical properties.

As these few examples show, gelatin nanofiber mats may have a broad range of physical and chemical properties, depending on the gelatin source, extraction, solvent, and electrospinning parameters, which are not always completely given in the literature. This has to be taken into account when evaluating the different crosslinking approaches as well as possible contradictory literature reports.

## 3. Physical Crosslinking

One of the possibilities to crosslink gelatin nanofibers is based on the use of high-energetic light, i.e., UV irradiation. In this approach, inter- and intramolecular photodimerization techniques are used to control the crosslinking density [29]. For example, Ko et al. firstly prepared trans-cinnamic acid modified gelatin, used it for electrospinning from HFP with a needle-based technique on a rotating collector, and crosslinked these gelatin nanofibers by UV irradiation at 254 nm for different durations, before the unreacted gelatin was rinsed with water [29]. They found higher UV absorbance with increasing amount of cinnamoyl groups, with no absorbance of natural gelatin at the investigated wavelength of 270 nm. The crosslinking density also increased with increasing UV irradiation duration up to 3 h, while longer times did not show further crosslinking effects. However, comparison with genipin crosslinked gelatin nanofibers showed higher cell proliferation of the latter.

An interesting effect of the UV irradiation duration was reported by Beishenaliev et al., who prepared gelatin by decalcification, thermosonication, and lyophilization of marine fish scales [30]. For needle-based electrospinning onto a stationary plate collector, the gelatin was dissolved in acetic acid/water in a ratio of 9:1. In contrast to Ko et al. [29], the photo-crosslinker was not added during electrospinning; instead, the nanofiber mats were placed in Petri dishes and irradiated from both sided at a wavelength of 254 nm using the UV crosslinker CL-508.G [30]. They found that depending on the UV irradiation duration between 5 and 20 min, the average fiber diameter was approximately double to triple the original value of approximately 50 nm. Unexpectedly, the longest UV irradiation time resulted in a completely dissolved nanofiber mat after 10 days of water resistance test, while the nanofiber mats crosslinked for 5 min or 10 min lost approximately 30% of their surface area after 14 days of incubation in medium. This was attributed to the molecular denaturation of the gelatin nanofibers during extended UV irradiation, as it had been reported before [31].

Ichimaru and Taguchi derived gelatin with molecular weight of 40,000 g/mol from Alaska Pollock [32]. They dissolved this gelatin in water/ethanol at 55 °C, added decanal for reductive amination of the amino groups and then 2-picoline borane to prepare decanyl-modified gelatin, which was electrospun from 50% ethanol aqueous solution at 55 °C in a needle-based process. Subsequent UV irradiation at 185 and 254 nm for 15–60 min was used to hydrophilize the nanofiber mat, as shown in previous reports [33,34]. UV irradiation significantly increased the burst strength and the remaining mass after 60–150 min in a collagenase solution [32]. It must be mentioned that crosslinking was here not the main goal of the UV treatment, so that no photo-crosslinking agent was applied.

One of the typical physical problems is the relatively low crosslinking extent, since only the surface is reached by these treatments, which requires dehydrothermal treatment (DHT), i.e., applying high temperatures under vacuum. Ratanavaraporn et al. combined this technique with different other physical and chemical crosslinking methods that were applied on nanofiber mats electrospun with a needle-based technique from gelatin type A and B in formic acid at different concentrations [35]. They found a high degree of crosslinking of nearly 80% for type B, which was treated by DHT at 140 °C for 48 h, and a lower degree of only approximately 55% for type A. Both values were higher than those for a pulsed inductively coupled plasma treatment under argon gas and significantly lower than all combinations of DHT with plasma treatment or chemical crosslinking.

**Figure 2:**
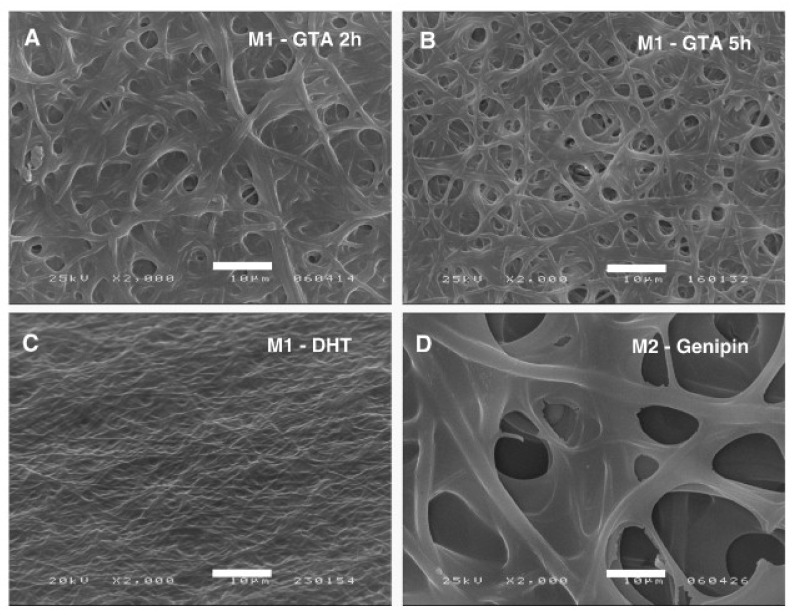
Scanning electron microscopy (SEM) images after immersion in Dulbecco’s modified Eagle medium (DMEM) for 3 days of: pure gelatin nanofiber mat crosslinked by (**A**) GTA 2 h; (**B**) GTA 5 h; (**C**) DHT; and (**D**) gelatin/genipin nanofiber mat (scale bar = 10 μm). Reprinted from [36]. Copyright 2021 Elsevier.

DHT of nanofibers prepared from gelatin from cold water fish skin was investigated by Gomes et al. [36]. The electrospinning solutions were prepared by acetic acid and distilled water, the latter also containing genipin. Electrospinning was performed with a needle-based technique on a slowly moving plate. For crosslinking, the samples were heated in an oven at different temperatures between 100 and 160 °C at a vacuum of 2 mbar for durations of 24–72 h. For the first solution, without genipin, DHT crosslinking for several days resulted in a reduction of the weight loss down to approximately 15%, while the genipin-containing nanofibers did not show a clear dependence on the crosslinking time. It should be mentioned that crosslinking with glutaraldehyde (GA) for more than 5 h could nearly fully avoid weight loss, showing that this chemical method is more efficient in this case. Figure 2 also shows that GA helped retain the nanofiber mat morphology to a certain amount, while the DHT-treated nanofiber mat was not dissolved but strongly modified. The genipin crosslinked nanofiber mats showed a fibrous structure with much larger fiber diameters [36]. Interestingly, in spite of the toxicity of GA, the cell viability after different cultivation times of 3T3 fibroblasts was acceptable or even better for gelatin nanofiber mats after 5 h of GTA, as compared to DHT treatment.

Ghassemi and Slaughter compared DHT with chemical crosslinking by EDS/Sulfo-NHS [37]. For type B gelatin, electrospun from acetic acid/distilled water by a needle-based technique, they found a DHT temperature between 120 and 180 °C to be optimal to build non-covalent bonds between gelatin molecules, with only small morphological changes due to crosslinking and slightly reduced average diameters. However, when these crosslinked gelatin nanofiber mats were immersed into medium (Dulbacco’s modified Eagle medium, DMEM) or phosphate-buffered saline (PBS) solution, they dissolved identically to the non-crosslinked nanofiber mat, showing that crosslinking here did not work well. In contrast, EDC/NHS crosslinking maintained their surface morphology in PBS, while they showed strong swelling in DMEM.

It should be mentioned that Mozaffari et al. report about the good crosslinking of gelatin type A nanofiber mats, electrospun from acetic acid and tannic acid, after applying a vacuum at a relatively low temperature of 45 °C [38], while DHT normally uses temperatures above 100 °C. Here, tannic acid supports chemical crosslinking [39,40,41] so that this vacuum treatment has no pure physical crosslinking method.

As these examples show, the non-toxic method of DHT crosslinking shows quite different efficiencies, depending on the gelatin type and the preparation conditions, and is due to its relatively low crosslinking effect mostly suitable for the area of drug delivery and other applications in which dissolution of the nanofiber mat within a defined time is required.

γ rays are high-energy electromagnetic radiation, which is typically produced by radioactive processes that are usually defined as having quantum energies of more than 200 keV or wavelengths of less than 5 pm. For example, they are used in medical radiation therapy, for sensory applications, sterilization, or the polymerization of materials. They can also be used for crosslinking gelatin. 

Since γ-sterilization is not unusual in biotechnology, crosslinking by γ rays also belongs to the physical techniques sometimes used to produce gelatin hydrogels [42,43]. However, studies about γ rays used to crosslink electrospun nanofiber mats were not found in the literature. This may be attributed to the fact that crosslinking by γ rays will produce hydrogels rather than completely avoid fiber swelling [44], making other crosslinking methods more attractive for the use with nanofiber mats.

**Figure 3:**
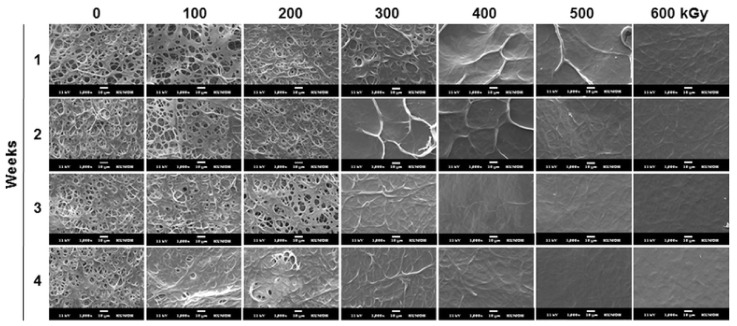
Morphology of e-beam crosslinked gelatin nanofiber mats as a function of irradiation dose and incubation period at *T* = 37 °C in PBS. Scale bars are 10 μm. Reprinted from [48], originally published under a CC-BY license.

Another crosslinking method, sometimes reported for gelatin, is electron beam irradiation [45,46,47]. Regarding nanofiber mats, Lee et al. used type B gelatin (250 bloom) for needle-based electrospinning from a TFE solution [48]. Parts of these nanofiber mats were crosslinked by glutaraldehyde; both treated and untreated nanofiber mats were afterwards e-beam treated at irradiations doses between 10 and 300 kGy (uncrosslinked gelatin) and between 100 and 600 kGy (GA treated gelatin), respectively, with dose rates of 8.33 kGy/s in all cases. They found a decrease of the molecular weight with increasing e-beam dose. Interestingly, e-beam irradiation here increased weight loss during incubation in PBS; i.e., it reduced the effect of the previous GA crosslinking step, as also visible in the morphology modification after incubation in PBS, as shown in Figure 3 [48]. Other attempts to use e-beam irradiation for crosslinking electrospun gelatin nanofiber mats were not found in the recent literature.

Finally, crosslinking gelatin by plasma treatment is sometimes reported [49,50]. For example, Liguori et al. used gelatin type A (300 bloom) from porcine skin for electrospinning core–shell fibers by a coaxial needle, in which the core contained genipin and the shell contained gelatin in acetic acid/water [51]. For comparison, pure gelatin nanofiber mats were electrospun with a common needle. These nanofiber mats were plasma treated using dielectric barrier discharge plasma in air at room temperature. They found no influence of the plasma treatment on both sorts of nanofiber mats. For both nanofiber mats, crosslinking by plasma treatment increased the mechanical properties and the morphological stability of the nanofiber mats in aqueous solutions, which was attributed to an increase of the crosslinked ε-amino groups, while additional stabilization without involving the gelatin free amino groups was found.

**Figure 4:**
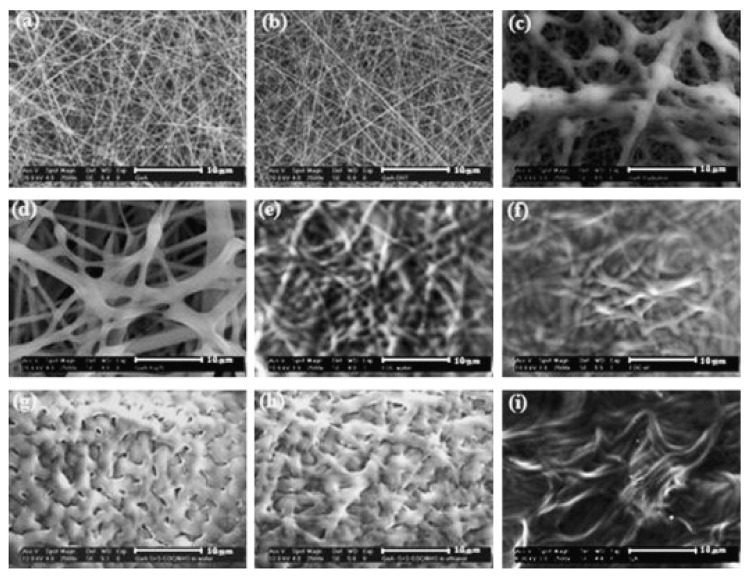
Scanning electron microscopy (SEM) images of electrospun gelatin type A nanofiber mats, crosslinked with different techniques (**a**) non-crosslinked, (**b**) DHT, (**c**) plasma, (**d**) DHT/plasma, (**e**) DHT/EDCw, (**f**) DHT/EDCe, (**g**) DHT/sEDCw, (**h**) DHT/sEDCe, and (**i**) DHT/vGA (scale bars = 10 μm). Reprinted from [35]. Copyright 2021 Elsevier.

Ratanavaraporn et al. used pulsed inductively coupled plasma in argon atmosphere at a pressure of 5 Pa, applying 1 pulse, as comparison to the aforementioned DHT treatment, as well as a combination of plasma and DHT treatment, and they found pure plasma treatment to show the lowest degree of crosslinking, while combining plasma with DHT showed a slightly smaller crosslinking degree for type B gelatin and a slightly larger value for type A [35]. It must be mentioned that different crosslinking techniques result in significantly different modifications of the nanofiber mat morphology, as depicted in Figure 4 [35].

Again, similar to the aforementioned physical crosslinking techniques, plasma treatment of gelatin nanofiber mats is not often found in the literature. In most cases, UV treatment or DHT are applied if physical crosslinking of gelatin nanofibers is desired. While only a few so-called negative results are reported, showing that transferring the physical crosslinking techniques applied on gelatin bulk materials is not necessarily successful (e.g., [48]), it can be assumed that more experiments have been performed and not published. In this area of research, more investigations are necessary, including clear reports on approaches that do not work, to allow researchers to find out whether alternatives to the often toxic chemical crosslinkers are available.

## 4. Chemical Crosslinking

**Figure 5:**
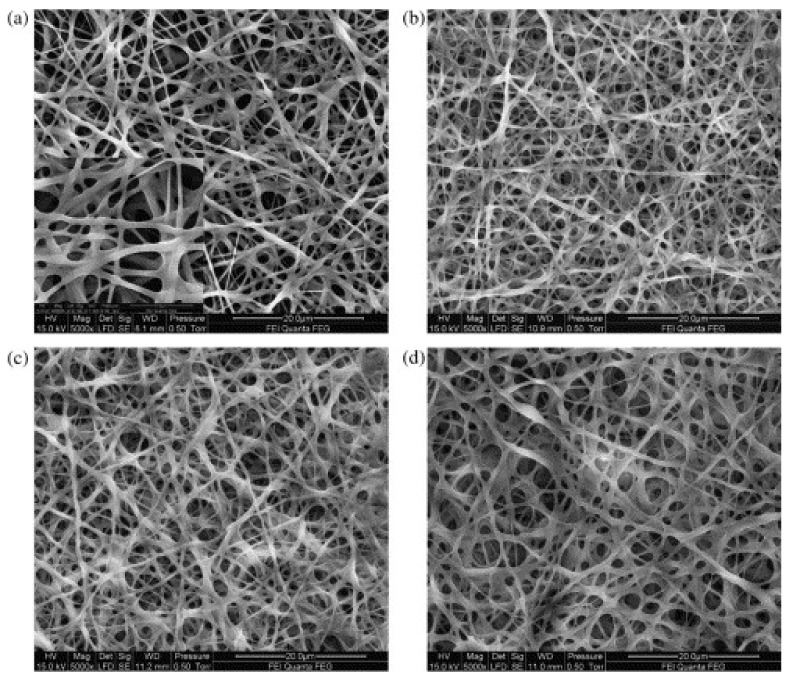
Crosslinked electrospun gelatin fibers: (**a**) before water-resistant test; immersed in 37 °C deionized water for (**b**) 2 days, (**c**) 4 days, and (**d**) 6 days (**d**). Reprinted from [53]. Copyright 2021 Elsevier.

The most often used chemical crosslinking agents belong to the aldehydes. In many studies, glutaraldehyde is used to crosslink electrospun gelatin nanofibers, either as vapor or in fluid form [52,53,54,55,56]. On the one hand, GA is a very efficient crosslinking agent, as visible in Figure 5 [53]. On the other hand, glutaraldehyde is toxic, with highly problematic effects on the human health, such as chronic bronchitis and even possible genetic activity, reported in diverse studies [57,58,59]. Thus, different crosslinking chemicals are necessary, combining low-toxicity with efficient crosslinking.

Another crosslinking approach for gelatin nanofiber mats is based on the aforementioned EDC/NHS. The idea behind this combination is that EDC promotes isopeptide bonds between the amino acid chains in proteins in an aqueous solution, while NHS subsequently stabilizes the EDC-fixed proteins [12,60].

For example, Agheb et al. compared chemical crosslinking by GA and EDC/NHS for electrospun nanofiber mats from modified gelatin–tyrosine by either integrating one of the crosslinkers into the gelatin solution or by crosslinking after electrospinning [61]. Interestingly, they found interconnected pores in the EDC/NHS crosslinked scaffold, while crosslinking with GA closed the pores. Both nanofiber mats crosslinked by integrating the chemicals into the spinning solution were found to be superior to the others, and after crosslinking, no cell-toxic residues were found in any of the nanofiber mats. Similarly, Hajiabbas et al. found increased mechanical properties and stability of gelatin nano fiber mats that were in situ crosslinked with EDC/NHS as well as no toxic effects on a cell culture [62].

Ghassemi and Slaughter compared DHT, genipin-EDC/Sulfo-NHS, and EDC/Sulfo-NHS crosslinked gelatin scaffolds for their possible use as three-dimensional scaffolds [63]. They used gelatin type B from bovine skin for needle-based electrospinning from an acetic acid/distilled water solution, alternatively with additional genipin in a solution of ethanol and PBS. For crosslinking, different concentrations of EDC were applied with 90% ethanol and various molar ratios of EDC/Sulfo-NHS. DHT was performed at 160 °C for 48 h at a vacuum of 22 mm Hg. While DHT crosslinking, as reported above from other studies, led to destroying the morphology upon immersion of the nanofiber mat in PBS or cell culture medium, degradation investigations after 14 days and 30 days of storage under different conditions showed the long-term stability of the EDC/Sulfo-NHS crosslinked nanofiber mats.

These samples underline the suitability of EDC/NHS crosslinking for gelatin nanofiber mats. This method is additionally known to enable producing non-toxic crosslinked collagen products [64]. EDC is normally regarded as non-cytotoxic and biocompatible [65], while both EDC and NHS can cause skin, respiratory, and serious eye irritations; i.e., they are not completely non-toxic. Too high concentrations of EDC/NHS may cause a certain risk of cytotoxicity [66], which is why ideally only low concentrations of this crosslinking combination are used [67].

Another approach that was mentioned in brief before is crosslinking gelatin nanofiber mats by different oxidized phenolic compounds. These crosslinkers are assumed to be safe and non-toxic. Applied in oxidizing media, the oxidation of phenolics to ortoquione and further reaction with amino or sulfhydryl groups within the protein structure result in strong C-N or C-S bonds [68], while in other environments, free radicals can be formed reacting with tyrosine, lysine, or cysteine so that protein molecules are crosslinked [69]. 

**Figure 6:**
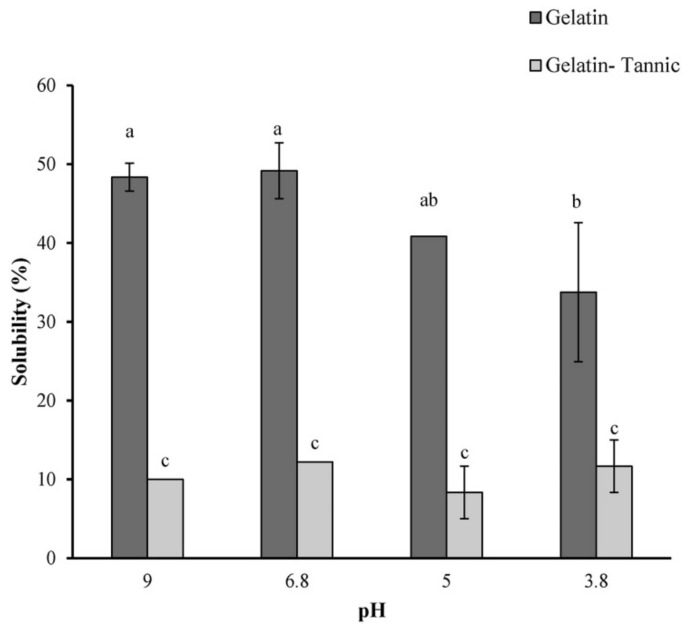
Solubility of pure and tannic acid-crosslinked gelatin nanofiber mats at different pH values. Different letters above column shows significant differences at *p* < 0.05. Reprinted from [70]. Copyright 2021 Elsevier.

Tavassoli-Kafrani et al. investigated tannic, gallic, ferulic, and caffeic acids as possible crosslinking agents for electrospun gelatin nanofibers [70]. They showed that especially tannic acid can be used as a crosslinking agent for gelatin nanofiber mats, as visible in Figure 6 from the strongly reduced solubility after crosslinking, and at the same time, it adds antioxidant and antimicrobial properties. The same group showed more recently that crosslinking gelatin nanofiber mats loaded with essential oil was also possible with tannic acid [71].

Gelatin type A from porcine skin was electrospun from acetic acid/water in a needle-based apparatus and crosslinked by tannic acid added to the gelatin solution. Mozaffari et al. found increased tensile strength and reduced elongation at break due to crosslinking, uniform nanofiber morphology for optimized spinning parameters, as well as antibacterial activity against *Staphylococcus aureus* and *Escherichia coli* [41].

As these few examples show, phenolics are highly interesting natural crosslinking agents. Due to their intrinsic antimicrobial properties, they may have an additional advantage in some applications, as compared to other chemical, physical, or enzymatic crosslinking agents.

**Figure 7:**
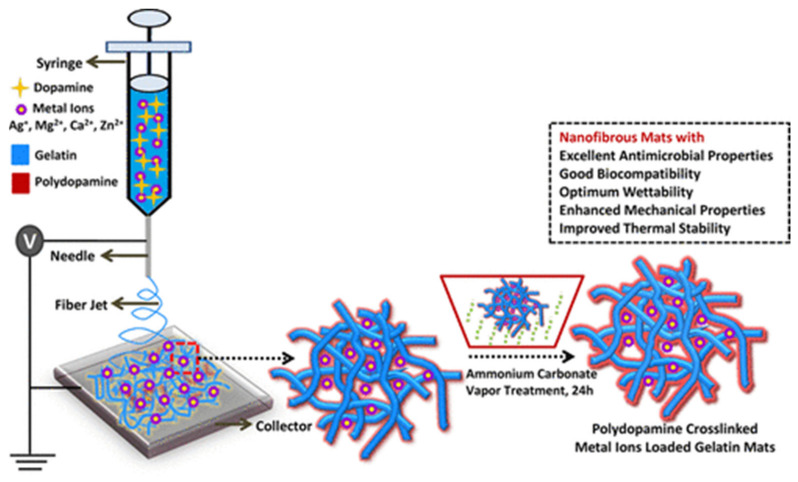
Electrospinning and crosslinking of metal-ion loaded gelatin nanofiber mats. Reprinted with permission from [72]. Copyright 2019 American Chemical Society.

Another nature-inspired crosslinker is polydopamine (pDA), which is an adhesive protein secreted by marine mussels. Leung et al. prepared antimicrobial nanofiber mats by needle-based electrospinning of a gelatin/pDA solution including different metal ions (Figure 7), which they treated by ammonium carbonate vapor to reach pDA-mediated crosslinking, which was improved by most of the metal ions [72]. Composite nanofiber mats including Ca^2+^ or Zn^2+^ ion were fond sterile against methicillin-resistant *S. aureus* and vancomycin-resistant *Enterococcus faecium* strains, while Ag^+^ ions led to a broader antibacterial activity against both Gram-positive and Gram-negative bacteria for a minimum of 40 days.

Similarly, Dhand et al. used polydopamine to crosslink gelatin type A (from porcine skin) nanofiber mats, which were electrospun in a needle-based technique from TFE [73]. Some electrospinning solutions were doped with dopamine, partly with different antibiotics, and tested against diverse Gram-positive bacteria, Gram-negative bacteria, and yeast strains. Dopamine crosslinking was performed in a desiccator with ammonium carbonate vapor. Measuring the inhibition zones against the different bacteria and yeast, they found high antimicrobial activity for most of the antibiotics over 20 days, showing that polydopamine crosslinking did not interfere with burn wound healing. While there are also studies reporting aqueous alkaline pDA coating methods [74,75,76,77], the studies shown here, working with the sublimation of ammonium carbonate, i.e., with an alkaline vapor phase in which the oxidative polymerization of dopamine is supported [78,79], seem to be advantageous in terms of retaining the original fiber morphology.

As these examples show, polydopamine-based crosslinking in an alkaline environment offers the possibility to prepare stable gelatin nanofiber mats without significant modifications of the morphology of the as-spun nanofiber mats.

Another quite often used crosslinking agent is glyoxal, which is the smallest dialdehyde (i.e., it contains two aldehyde groups). It is less toxic than the highly toxic formaldehyde and glutaraldehyde; however, it can also cause skin, respiratory, and serious eye irritations. Opposite to the aforementioned EDC and NHS, the ability to cause genetic defects cannot be excluded; thus, while often used as a crosslinker for gelatin [55,80,81,82], glyoxal will not be treated in detail here. The same is valid for epichlorohydrin, which can cause skin corrosion, is acutely toxic for oral or dermal contact or on inhalation, and is carcinogenic, but nevertheless is regularly used as a crosslinking agent for gelatin [83,84,85].

However, there are some other materials that are non-toxic or at least low-toxic, or the toxicological properties were not investigated. Proanthocyanidin, also called procyanidine, a natural plant metabolite, is one of these materials that is sometimes used as a crosslinking agent for gelatin [86,87,88]. Huang et al. report on crosslinking gelatin nanofiber mats by proanthocyanidin; however, they performed electrospinning with a needle-based process from gelatin/PVA/proanthocyanidin dissolved in formic acid, while the actual crosslinking step was performed in glutaraldehyde [89]. Chen et al. crosslinked gelatin type A (300 bloom), electrospun from TFE in a needle-based process, by GA, genipin, and also procyanidine [90]. They found a temperature-dependent redshift of the amide I absorption band in the infrared spectrum of the procyanidine crosslinked nanofiber mats, which they attributed to the temperature dependence of the intra-/intermolecular hydrogen-bonding of gelatin; i.e., it disrupted hydrogen bonding interactions at higher temperatures and thus increased interactions between procyanidine and gelatin. Compared with GA and genipin, crosslinking with procyanidine resulted in the highest elastic moduli, and the remaining mass after enzymatic degradation during 96 h was highest. The morphology after 1 d or 2 d of enzymatic degradation was also superior for procyanidine crosslinking, showing that procyanidine crosslinking is indeed a highly interesting method. Nevertheless, more reports dealing with crosslinking gelatin nanofiber mats by procyanidine were not found in the literature.

Another class of substances sometimes used for the crosslinking of gelatin is poly(carboxylic acids), i.e., dicarboxylic acids with unbranched chains, containing two COOH groups. Some examples are oxalic acid, citric acid, fumaric acid, or maleic acid. While fumaric acid is usually regarded as low-toxic, oxalic acid is corrosive, and citric acid is skin and eye irritant. For citric acid, the crosslinking effect was attributed to reactions between the carboxylic groups of the citric acid and the amino groups of gelatin, as shown by infrared spectroscopy [91]. Crosslinking gelatin films with citric acid was performed by mixing both materials with agar and glycerol, heating, and pouring into Petri dishes [92,93]. One study about crosslinking electrospun nanofiber mats with citric acid did not report successful crosslinking; water solubility was not significantly reduced [94].

Finally, saccharides can be used to crosslink gelatin, based on the so-called Maillard reaction, which starts with a condensation reaction between saccharides and amino acids and, along different pathways, finally results in irreversible crosslinking [94]. Etxabide et al. report on a significantly reduced solubility of fish gelatin-based films especially for ribose as a crosslinker, while glucose and lactose also showed an effect after 6–24 h of heat treatment [95]. Siimon et al. investigated the crosslinking of nanofiber mats prepared by needle-based electrospinning of gelatin type A from porcine skin with glucose and glacial acetic acid, which was followed by thermal treatment [96]. They found too high glucose concentrations to make the nanofiber mats brittle, while the elastic modulus could be increased by crosslinking.

**Figure 8:**
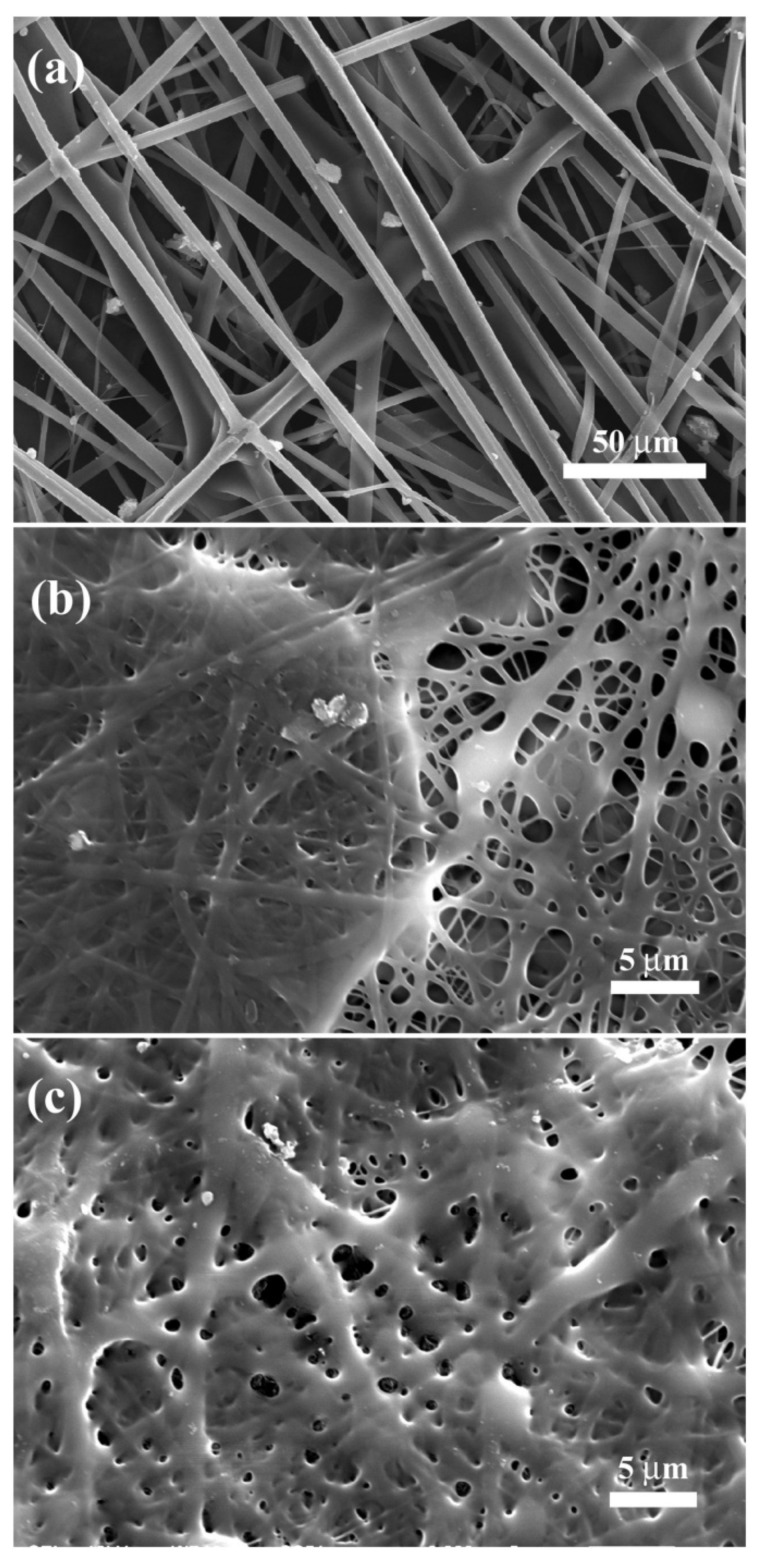
SEM images of the electrospun nanofiber mats produced from (**a**) pure gelatin; (**b**) gelatin/glycerol; (**c**) gelatin/glycerol/glucose. Reprinted from [97]. Copyright 2021 Elsevier.

Morsy et al. combined glucose with glycerol, both of which can react with the amino acids in gelatin via the Maillard reaction, as crosslinkers in the electrospinning solution of gelatin type A in glacial acetic acid [97]. To enhance crosslinking, they firstly prepared an aqueous solution of gelatin and glycerol, heated it up to 90 °C, cooled it down to room temperature, and dried it at 40 °C for 72 h to form gelatin–glycerol powder, which was dissolved in glacial acetic acid to prepare an electrospinning solution. Partly, glucose was added to this solution. The electrospun nanofibers mats show strong modifications of the surface morphology as compared to pure gelatin nanofiber mats (Figure 8), water uptake of up to more than 800% after 10 days, and degradation after 10 days (gelatin/glycerol) or 14 days (gelatin/glycerol/glucose), respectively.

Most recently, Kwak et al. used sucrose, glucose, and fructose to crosslink cold water fish gelatin, which was needle-electrospun from distilled water with one of the sugars added [98]. After spinning, crosslinking was performed at a temperature of 100 °C for 4 h. They found the highest crosslinking degree for fructose, followed by glucose, while sucrose had nearly no impact. Similarly, the tensile stress and Young’s modulus were most enhanced by fructose, while the maximum strain at break was largest for the sucrose-crosslinked nanofiber mat. Only after fructose crosslinking, the sample mass remained nearly unaltered after 10 days of hydrolytic degradation.

A higher temperature of 170–175 °C, applied for 3 h was chosen by Siimon et al., who used gelatin type A from porcine skin, gelatin type B from bovine skin, and glucose in glacial acid for needle-based electrospinning of nanofiber mats [99]. Depending on the glucose content, different modifications of the nanofiber mat morphologies were already found before thermal crosslinking. Chemical investigations showed an optimum glucose content of 20%. For higher glucose concentrations, the number of viable fibroblast cells seeded on these nanofiber mats was lower. On the other hand, scaffolds with higher glucose contents of 25% and 30% showed resistance to digestion in collagenases and trypsin, while 20% glucose resulted in partial degradation and lower glucose content in full dissolution.

As these examples show, promising approaches to reach chemical crosslinking of gelatin nanofiber mats are mostly based on EDC/NHS, phenolics such as tannic acid, polydopamine, or saccharides. On the other hand, there are still diverse approaches that have not been optimized or not even been tested on gelatin nanofiber mats. Here, more research is necessary to find more possible chemical crosslinkers that work also on nanofibrous materials.

## 5. Enzymatic Crosslinking

Enzymatic crosslinking of gelatin is most often performed by genipin [100,101,102] or microbial transglutaminase [103,104,105]. Both are not non-toxic, but they are briefly mentioned here as examples for enzymatic crosslinking. Genipin is a molecule extracted from the gardenia plant fruit, which crosslinks gelatin in a two-step process, firstly by building heterocyclic linking of genipin to an amine in gelatin, followed by a nucleophilic substitution of the ester group in genipin resulting in covalent crosslinks between the primary amine residues [101]. Microbial transglutaminase (MTG) is a transferase that is assumed to build covalent bonds between different polymer chains, resulting in a hydrogel network [105]. Other enzymes that have been used to crosslink biopolymers belong to the transferases, hydrolases, and oxidoreductases [105], and they have been scarcely investigated for their possible use with electrospun nanofiber mats.

**Figure 9:**
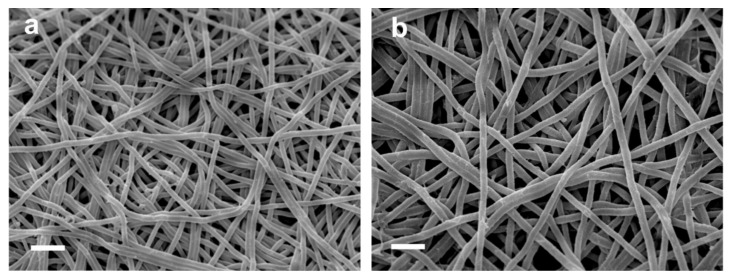
SEM images of nanofiber mats crosslinked by genipin, followed by PBS rinsing, spun from (**a**) gelatin; (**b**) gelatin/genipin, after 7 days immersion in DMEM. Scale bars depict 5 µm. Reprinted from [106]. Copyright 2021 Elsevier.

Panzavolta et al. investigated crosslinking nanofiber mats from type A gelatin from porcine skin, which were electrospun with a needle-based system from acetic acid/distilled water, purely or with additional genipin after an incubation time of 30 min [106]. Afterwards, they were soaked in genipin solution in ethanol at different concentrations and crosslinking times, whereafter they were partly rinsed in PBS and dried at different temperatures. They found that PBS rinsing after the genipin treatment was necessary to retain the fiber morphology, as depicted in Figure 9. With this optimized method, more than 90% of crosslinking could be reached, in contrast to the as-spun gelatin/genipin nanofibers showing values around 15%. Interestingly, no large differences between gelatin and gelatin/genipin nanofiber mats were observed in terms of morphology or mechanical properties.

Su and Mo used a genipin vapor at room temperature instead to crosslink electrospun gelatin nanofiber mats [107]. Depending on the crosslinking conditions, they found that the time-dependent release of a model drug made these nanofiber mats suitable for drug delivery, while they could also suit as tissue engineering scaffolds for a higher degree of crosslinking. Del Gaudio et al. loaded human vascular endothelial growth factor into electrospun gelatin nanofiber mats crosslinked with two different genipin concentrations, and they found a cumulative release of approximately 60–90% after 28 days, depending on the crosslinking degree [108].

Another method was suggested by Gualandi et al., who used coaxial electrospinning to mix gelatin and genipin solutions during spinning, with the possibility to tailor the genipin content by varying the feeding rates of the solutions [109]. After a thermal treatment for 1–3 days, followed by rinsing in ethanol and PBS, highly crosslinked gelatin nanofibers were produced with retained morphology in aqueous solution and increased mechanical properties.

In spite of these advantageous properties of genipin for crosslinking gelatin, it must be mentioned that it is toxic if swallowed and thus is only mentioned here as one of the few examples of enzymatic crosslinking found in the literature. Microbial transglutaminase (mTG), on the other hand, may cause breathing difficulties, allergies, or asthma when inhaled and is thus less toxic, but it is still far from being harmless, while it is nevertheless cytocompatible.

Although mTG is often found in the literature as a crosslinker for gelatin, studies can only be found on pure gelatin, which is not electrospun or on electrospun gelatin blends [110,111,112,113,114]. Apparently, the topic of crosslinking electrospun gelatin nanofibers needs to be investigated in more detail in the future.

## 6. Blending Gelatin with Other Polymers

As a last method to improve the mechanical strength and to reduce the water solubility of electrospun gelatin nanofiber mats, they can be blended with other polymers, in this way often retaining the positive properties of gelatin for tissue engineering in combination with the mechanical stability of the water-stable polymer. 

One of the typical blend partners of gelatin is chitosan. Chitosan is derived from chitin by partial deacetylation, and it is, similar to gelatin, biocompatible, biodegradable, and antimicrobial [115]. Dhandayuthapani et al. prepared chitosan/gelatin blend nanofibers by electrospinning from TFA/dichloromethane solution and found significantly better tensile strength, in the range of normal human skin, for the blended nanofibers [116]. Similarly, Yin-Guibo et al. found improved mechanical properties for silk fibroin/gelatin blended electrospun nanofiber mats, as compared to both pure materials [117]. The solubility in water was not mentioned in these studies.

**Figure 10:**
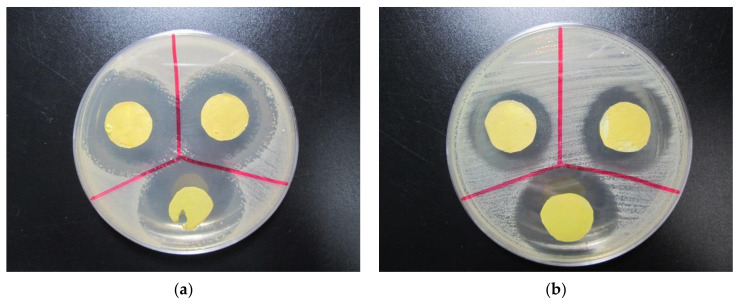
Antibacterial activity of 14% *w*/*v* PCL/GE-based nanofiber against (**a**) *Bacillus cereus*; (**b**) *Escherichia coli*. From [118], originally published under a CC-BY license.

The latter can be improved by blending gelatin with water-resistant polymers, such as polycaprolactone (PCL), which is a synthetic polymer often used in tissue engineering. Chong et al. reported on PCL/gelatin blend nanofibers electrospun from formic acid in a needle-based technique and loaded with an antibiotic model drug [118]. They showed a strong increase of the average fiber diameter with the polymer concentration between 12% and 18%, an increase of the hydrophilicity with increasing gelatin content, and a weight loss of up to 16% for the blend fibers after 14 days, while no weight loss occurred for the pure PCL fibers. Antibacterial tests against Gram-positive *Bacillus cereus* and Gram-negative *E. coli* showed large inhibition zones, as visible in Figure 10.

Kim et al. blended gelatin with polyurethane (PU) in different ratios to electrospin nanofiber mats from a hexafluoro-2-propanol solution for wound healing [119]. All ratios between 100% gelatin and 100% PU were spinnable. Contact angles decreased and water uptake increased with increasing gelatin content, and degradation in PBS solution at 37 °C showed more remaining mass after 1–5 weeks for lower gelatin contents, while all samples containing gelatin showed significant morphological changes. On the other hand, the adhered cells stretched more easily on samples with higher gelatin content, and proliferation rates were significantly increased with increasing gelatin content.

Similarly, blending gelatin with poly(lactic acid) (PLA) led to increased water stability, as compared to pure gelatin, and on the other hand significantly higher hydrophilicity and cell viability after 3 d and 7 d, as compared to pure PLA [120,121]. Similarly, blending gelatin with poly(D,L-lactide-co-glycolide) (PLGA) resulted in a higher swelling ratio than in pure PLGA nanofibers, larger fiber diameters with higher PLGA content, and again improved adhesion and proliferation of cells for the blend fibers as compared to pure PLGA nanofibers [122]. Electrospun gelatin/polyacrylonitrile (PAN) nanofiber mats were found to be not only well suited as a precursor of carbon nanofibers with increased fiber diameters due to adding gelatin to PAN [123] but also as scaffold for mammalian cell growth [124,125]. Blending gelatin with a copolymer of poly(glycerol sebacate) (PGS), a biodegradable and elastic polymer, and poly(methyl methacrylate) resulted in good hydrophilicity and biocompatibility due to gelatin as well as a high proliferation of rat PC12 cells on these electrospun nanofiber mats [126]. For electrospun gelatin nanofiber mats blended with poly(l-lactic acid)-b-poly(*ε*-caprolactone) (PLLCL) in different ratios, 40 wt % PLLCL was found to be optimum in terms of cell proliferation, and at the same time, it showed suitable tensile stress in dry and wet conditions [127].

As these examples show, blending gelatin with different water-stable polymers offers many possibilities to combine the desired mechanical and hydrophilic properties to prepare scaffolds well suitable for tissue engineering and generally the growth of mammalian cells.

## 7. Biomedical Applications of Crosslinked Gelatin Nanofiber Mats

In addition to the aforementioned possible applications of crosslinked gelatin nanofibers mats for tissue engineering, cell growth, wound healing, and other biomedical applications, this section gives some more examples of these applications without the previous restriction to non-toxic crosslinking methods.

Jalaja et al. used crosslinking with dextran aldehyde to improve thermal and mechanical properties as well as the structural integrity in an aqueous solution [128]. The nanofiber mat was found to be non-cytotoxic for mouse subcutaneous fibroblasts L-929 and showed significantly higher cell proliferation as compared to glutaraldehyde crosslinked nanofiber mats.

Using GT vapor crosslinking, Gui et al. compared human mesenchymal stem cell growth on randomly oriented and highly aligned gelatin nanofiber mats and found more effective proliferation on the oriented fibers [129]. Padrao et al. also used GA to crosslink electrospun gelatin nanofiber mats and found no inhibition of MC-3T3-E1 cell adhesion and proliferation in randomly oriented and aligned gelatin nanofiber mats [130]. With genipin crosslinking of gelatin nanofiber mats, Gualandi found no cytotoxicity against human primary chondrocytes, but oppositely the promotion of chondrocyte viability and differentiation [109]. Chen et al. also found cell compatibility for mouse mesangial cells grown on GA-crosslinked gelatin nanofibers [56]. 

Luo et al. compared gelatin nanofiber mats crosslinked with glutaraldehyde, genipin, and EDC/NHS and found that they all supported adhesion, spreading, and proliferation of MC-3T3-E1 cells, with a lower cell viability after GA crosslinking, while similar values were found for genipin and EDC/NHS [131]. 

Ghassemi and Slaughter used DHT and EDC/NHS crosslinking on gelatin nanofiber mats and suggested EDC/NHS crosslinked electrospun gelatin nanofiber mats as scaffolds for cell-based assays [37].

Hivechi et al. used NIH/3T3 mouse fibroblasts to investigate the influence of adding cellulose nanocrystals to electrospun gelatin nanofibers [132]. They found a slightly increased biodegradability of the fibers crosslinked by GA vapor and no effect on the cytotoxicity, cell growth, and proliferation. 

Using GA vapor crosslinking, Laha et al. used gelatin nanofiber mats for oral drug delivery with piperine as a model drug [52,133]. By modulating the pH value of the release medium and the crosslinking efficiency, they could tailor the piperine release rate. 

Del Gaudio et al. also used genipin-crosslinked electrospun gelatin nanofiber mats to investigate loading with human vascular endothelial growth factor (VEGF) and long-term release of VEGF [108]. Human mesenchymal stromal cells growing on these nanofiber mats showed higher cell viability and endothelial differentiation when growing on these VEGF-loaded nanofiber mats, with the bioactive and pro-angiogenic potential kept for two weeks, showing that VEGF-loaded genipin-crosslinked gelatin nanofiber mats may be suitable for the stimulation of angiogenesis in tissue engineering.

Bactericidal wound dressing was produced by adding poly([2-(methacryloyloxy)ethyl] trimethylammonium chloride) (PMETAC) to the gelatin electrospinning solution and crosslinking the obtained nanofiber mats with GA vapor [134]. Inal and Mülazimoglu showed good cell attachment and proliferation and suggested using these antimicrobial, biocompatible nanofiber mats as wound dressing.

Especially for postoperative surgical wounds, Rath et al. suggested gelatin nanofiber mats loaded with cefazolin/zinc oxide nanoparticles as antimicrobial wound dressings [135].

As these few examples show, in addition to those given in the previous sections, the main applications of crosslinked gelatin nanofiber mats in biomedicine are related to tissue engineering and cell growth, but also for wound healing with tailorable drug delivery.

## 8. Conclusions and Outlook

Gelatin can be crosslinked to reach a defined water-stability and desired mechanical properties by physical, chemical, or enzymatic methods as well as by blending with other polymers. However, many of these methods are only scarcely investigated or not reported at all for gelatin nanofiber mats. Especially the low-toxic or non-toxic crosslinkers mostly necessitate more research to develop their full potential for crosslinking electrospun nanofiber mats. Recently, none of the describe methods can be mentioned as the optimum one, while some chemical methods such as EDC/NHS, phenolics such as tannic acid, polydopamine, or saccharides provide already relatively good combinations of crosslinking degree and low toxicity. A brief overview of water stability and crosslinking efficiency for different methods is given in Table 1.

As this review shows, more research is necessary to transfer more methods from crosslinking of macroscopic gelatin to nanofiber mats, especially due to the well-known problem that nanofiber mats tend to containing a higher amount of water than bulk materials due to their large specific surface, which may necessitate additional pretreatments or crosslinking in a vacuum chamber. On the other hand, even this relatively small amount of presented studies often showed contradictory results, which are apparently based on different types and sources of gelatin, while other parameters, such as the solvent chosen for electrospinning, the porosity of the nanofiber mat, etc. may also have a significant impact. This shows that also many more systematic studies are necessary, varying one of these parameters and investigating its impact on the crosslinking efficiency. It should be also mentioned that images of the nanofiber mat morphology after inserting a crosslinked mat in a fluid are highly encouraged, since some of the examples given here show strong morphological modifications in addition to a loss of mass.

## Figures and Tables

**Table 1:** Effect of crosslinking for different crosslinking techniques after electrospinning.

Electrospinning Process	Crosslinking	Effect	Ref.
Marine fish-scale gelatin spun from acetic acid/water	UV crosslinker added in petri dish, UV irradiation at 254 nm for 5–20 min	30% surface area lost after 14 day in medium	[30]
Alaska Pollock gelatin spun from water/ethanol at 55 °C	UV irradiation at 185 nm and 254 nm for 15–60 min without crosslinker	Increased burst strength and remaining mass after 60–150 min in collagenase solution	[32]
Gelatin type A and B spun from formic acid	Dehydrothermal treatment (DHT)	80% efficiency for type B, 55% for type A	[35]
Cold water fish skin gelatin spun from acetic acid/water	DHT	Weight loss reduced to 15%, modified fiber morphology	[36]
Type B electrospun from acetic acid/distilled water	DHT	Immersion in PBS or DMEM dissolved fibers	[37]
Type A from porcine skin spun from acetic acid/water	Dielectric barrier discharge plasma in air at room temperature	Increased mechanical properties and morphological stability in aqueous solution	[51]
Gelatin type A and B spun from formic acid	Pulsed inductively coupled plasma in argon atmosphere at 5 Pa	Lower degree than DHT	[35]
Type B from bovine skin spun from acetic acid/water	EDC/NHS	Long-term stability in PBS and medium	[63]
Type A from porcine skin spun from acetic acid/water	Tannic acid	Increased tensile strength, reduced elongation at break	[71]
Type A from porcine skin from 2,2,2-trifluoroethanol (TFE)	Polydopamine + ammonium carbonate vapor	Good retaining of the original fiber morphology	[73]
Type A spun from TFE	Proanthocyanidin (procyanidine)	High remaining mass after enzymatic degradation and retaining morphology well	[90]
Type A spun from glacial acetic acid	Glucose and glycerol	Strong modifications of surface morphology, reduced degradation in water	[97]
Cold water fish gelatin spun from water + sugar	Fructose and other sugars at 100 °C for 4 h	Nearly unaltered sample mass after 10 days for fructose crosslinking	[98]
Type A from porcine skin sun from acetic acid/water	Genipin	>90% for optimized parameters	[106]

## Data Availability

No new data were produced in this review paper.
